# The effectiveness of systemic antibiotics for osteomyelitis of the foot in adults with diabetes mellitus: a systematic review protocol

**DOI:** 10.1186/s13047-022-00554-3

**Published:** 2022-06-17

**Authors:** Akram Uddin, David Russell, Fran Game, Derek Santos, Heidi J. Siddle

**Affiliations:** 1 Northamptonshire Healthcare NHS Foundation Trust, Essex Partnership University NHS Foundation Trust & University of Nottingham, Nottingham, UK; 2 Department of Podiatric Surgery. Danetre Hospital, London Road, Northamptonshire, NN11 4DY UK; 3 Leeds Institute of Clinical Trials Research, University of Leeds (and Leeds Vascular Institute, Leeds Teaching Hospitals NHS Trust), Leeds, LS2 9JT UK; 4 University Hospitals of Derby & Burton NHS Foundation Trust, Derby, UK; 5 Queen Margaret University, Edinburgh, UK; 6 Institute of Rheumatic and Musculoskeletal Medicine, University of Leeds, Leeds, UK

**Keywords:** Osteomyelitis, Bone infection, Diabetic foot, Diabetic foot infection, Systemic antibiotics, Oral antibiotics, Intravenous antibiotics

## Abstract

**Background:**

Osteomyelitis of the foot is a major complication of diabetes that can be limb and life threatening. Systemic antibiotic pharmacotherapy is often used first line to eradicate infection and allow restoration of devitalised bone.

The aim is to conduct a systematic review of the effectiveness of systemic antibiotics on osteomyelitis of the foot in adults with diabetes mellitus.

**Methods:**

A systematic review of all interventional studies treating osteomyelitis with systemic antibiotics in participants with diabetes mellitus and an ulcer of the foot below the malleoli will be conducted. Studies not available in English and in people below the age of 18 will be excluded. Study selection will follow the Patient Reporting Items for Systematic Reviews and Meta-Analysis (PRISMA-P guidelines). The quality of the studies will be assessed using the Cochrane risk-of-bias tool (RoB 2) for all randomised controlled trials and the Newcastle–Ottawa Scale (NOS) will be used for non-randomised controlled trials. Electronic databases will be searched with no timeline restrictions.

**Data Extraction:**

All identified references will be imported to the Rayyan Application. Studies for eligibility will be screened by two reviewers. One reviewer will perform the data extraction and quality appraisal will be conducted by two authors. If sufficient data is available, the quality will be analysed and a meta-analysis will be performed. Data synthesis will be conducted, and meta-analysis undertaken using RevMan 5.4.1 Meta-analysis software. Non-parametric data may be compared between selective intervention and outcomes.

**Discussion:**

The results of this systematic review will identify the effectiveness of systemic antibiotic therapy on osteomyelitis of the foot in people with diabetes based on the set outcome measure criteria. The findings will establish if there are existing consistent standards or variation in practice when treating diabetic foot osteomyelitis (DFO). The study may establish if guidelines are required to standardise practice when treating DFO with systemic antibiotic therapy. This systematic review protocol will synthesise the existing evidence on the effectiveness of systemic antibiotic therapy for treating DFO.

**Trial registration:**

International Prospective Register for Systematic Reviews (PROSPERO) number CRD42021245424.

**Supplementary Information:**

The online version contains supplementary material available at 10.1186/s13047-022-00554-3.

## Background

Infection of the foot in people with diabetes mellitus can lead to significant morbidity and mortality [1–5]. The contribution of hyperglycaemia, peripheral neuropathy, vascular insufficiency or trauma in a patient with diabetes mellitus can lead to the development of diabetic foot ulcer (DFU) which may become the point of entry for pathogens [6–8]. It is estimated the lifetime risk of a person with diabetes developing a DFU is as high as 25% and infection complicates these in 40–80% [9]. Diabetic foot osteomyelitis (DFO) is the consequence of a soft tissue infection that progresses into bone by breaching the cortex and invading the medullary cavity [10, 11]. It is estimated 20% of infected DFU will result in DFO [9]. Infected DFU in this patient group is associated with the use of prolonged antibiotic therapy, hospitalisation and surgery [9]. Increased healthcare costs, adverse drug reactions and antibiotic resistance are associated with overuse of antibiotics [12].

The Infectious Disease Society of America (IDSA) and International Working Group on the Diabetic Foot (IWGDF) classify diabetic foot infection (DFI) and DFO based on clinical presentation [13, 14]. These clinical signs may include inflammation, purulent or non-purulent secretions, malodour and a positive probe-to-bone (PTB) [11, 13, 14]. These guidelines highlight the most appropriate diagnostic processes and treatment interventions that include antibiotic therapy for DFO. Although many antibiotics are used to treat DFO, the most appropriate practice has not been established [15]. Furthermore, it has been suggested that comparison of studies treating DFO is difficult due to differing diagnostic criteria and treatment regimens [16]. It has not been established whether the optimal treatment of DFO is surgical intervention in conjunction with appropriate antibiotics or by systemic antibiotic pharmacotherapy alone [17]. Some authors have suggested that surgical debridement is crucial in the treatment of DFO [8], whilst other studies have shown antibiotic pharmacotherapy alone to be sufficient when treating DFO [18].

The most common pathogens involved in DFO are aerobic gram positive staphylococcus aureus and streptococcus species [5, 8, 10, 16, 19, 20]. Gram negative pathogens including the Enterobacteriaceae family including Escherichia coli, Klebsiella pneumoniae, Morganella morganii and Proteus mirabilis have also been reported [8, 20]. Pseudomonas is regarded as a rare invader in non-humid climate countries and although infrequently isolated on wound swabs it is usually a coloniser and not a cause for diabetic foot infections [21, 22]. Anaerobes are reported to be more likely involved in necrotic wounds and gangrene [1].

A prolonged course of oral antimicrobial therapy may contribute to the evolution of antimicrobial-resistant bacteria and antibiotic related complications such as Clostridium difficile colitis [16, 23, 24]. The prevalence of antibiotic resistant and multiple drug resistance organisms (MDRO) and organisms isolated from people with diabetes has been reported to be increasing [1, 10]. A surgical approach and use of synthetic orthobiological agents combined with a reduced duration of systemic antibiotics for the management of DFO has been proposed to reduce these risks as well as achieving optimum concentrations of antibiotic at the site of infection [8, 25, 26]. The successful treatment of DFO with antibiotic therapy alone without surgery has been reported to be associated with microbiological assessment of bone samples [27]. The treatment choice for a presenting DFO is based on multiple factors but it is often the treating clinician who must weigh up the risks and benefits given the patients co-morbidities.

The diagnosis of DFO is recommended as above by IDSA and IWGDF guidance clinical, but in all cases microbiological samples should be taken to identify the pathogen involved to support targeted therapy [4, 5, 10, 11]. Microbiological samples include deep tissue and bone for culture and antibiotic sensitivity testing. Tissue samples are more specific for bacterial identification than superficial wound swabs alone [20]. Blood samples should also be taken for serum biomedical markers and haematological testing that may support a clinical diagnosis of infection [1, 5, 11, 28]. Diagnosis should also be supported by radiologic investigations [10, 29]. With technological advances, sophisticated imaging modalities such as contrast Magnetic Resonance Imaging (MRI), positive emission tomography (PET)/computed tomography (CT) and single-photon emission computed tomography (SPECT)/CT may also be considered subject to their availability. The use of radiolabelled autologous white blood cells (WBC) and anti-granulocyte antibodies (anti-G-mAb) is also a recognised practice in the identification of DFO [30]. A combination of diagnostic interventions are considered to be an appropriate approach to diagnose DFO [10].

Antibiotic regimens are initially empirical and may be based on the severity of presenting infection and local antibiotic policies, with targeted narrow spectrum agents introduced after positive culture and sensitivity results are available [5]. The aim of antibiotic therapy is to achieve high concentration of antibiotics at the site of infection [9]. In addition to data suggesting successful treatment of DFO with systemic antibiotics [16, 18], a more recent trial suggests there is no clinical outcome difference between oral and intravenous antibiotic therapy when treating osteomyelitis, although not all participants in this trial had diabetes [31]. Furthermore, the incidence of concurrent peripheral arterial disease (PAD) or critical limb threatening ischaemia (CLTI) remains unknown in this trial which may have caused discrepancies in optimum delivery or concentration of antibiotics to bone regardless of systemic antibiotic mode of delivery. There is no proven laboratory test or imaging modality to determine when antibiotic therapy should be discontinued [5]. The duration of antibiotic therapy is not consistent for treating DFO and studies have reported on similar outcomes when comparing 3 and 6 weeks or 6 weeks short term and 12 weeks long term antibiotic therapy [32, 33]. The optimum duration of antibiotic therapy for DFO is therefore not well defined [11].

There is existing evidence to hypothesise systemic antibiotic therapy treats DFO [16, 18, 34, 35]. However, some fail to treat DFO with reported worsening infection requiring surgical debridement or amputation [27, 36, 37]. Therefore, investigation is required to identify the most effective systemic antibiotic treatment for DFO.

A recently published systematic review has analysed the effectiveness of all interventions in the management of infection of the diabetic foot [3]. However, this systematic review did not specifically identify the outcome measures for DFO with systemic antibiotics [3]. A second systematic review by the same authors on the diagnosis of DFI does however provide some guidance for clinicians on clinical and inflammatory markers for the diagnosis of osteomyelitis [28]. The systematic review does not however identify the single or combined diagnostic investigations that should be considered to monitor progress or complete resolution when treating DFI or DFO with systemic antibiotics.

It is evident there remains uncertainty about the most appropriate antibiotic therapy for the management of DFO. The challenge is not to identify the most effective antibiotic but to establish the antibiotic intervention and practice that is most effective for the eradication of DFO and preservation of limb. A systematic review is therefore required to establish this.

We present a systematic review protocol that will review the effectiveness of systemic antibiotic therapy for DFO and identify outcomes used to determine the effectiveness. The systematic review will aim to identify the role of systemic antibiotics when treating DFO. To our knowledge there is no published systematic review addressing our proposed question.

## Methods

Details of this protocol are registered on the International Prospective Register of Systematic Reviews (PROSPERO) registry. The planned start date is from the inception of databases to the 30^th^ April 2022.

### Inclusion criteria for selected studies

#### Population

- Adults age ≥ 18 years or over.
- Diagnosed with diabetes mellitus (of any type).
- Diagnosed with osteomyelitis (by any means) distal to malleoli.
- Osteomyelitis caused by any micro-organism.

### Study design

The study design is a systematic review.

#### Intervention

- Randomised controlled trials (RCTs), quasi-experimental, cohort studies and case series that involve intervention by delivery of systemic oral or intravenous antibiotic therapy
- Studies that involve intervention by delivery of systemic oral or intravenous antibiotic therapy as part of a bundle of care i.e. surgery/debridement/drainage
- Systemic antibiotic intervention by a prescribing healthcare professional

### Comparator/control

Any patient/groups of patients who were not treated with systemic oral or intravenous antibiotic therapy alone.

#### Outcomes

The effectiveness of systemic antibiotic therapy determined by the resolution of DFO by:

- Blood tests
- Biomarkers including procalcitonin (PCT)
- Radiological imaging
- Healing of ulcer
- Limb preservation
- No recurrence of osteomyelitis
- Outcome grades/score
- Acute kidney injury (AKI)
- Minor or major amputation
- Mortality

### Exclusion Criteria

Studies will be excluded based on the following criteria:

- Adults below age of 18 years.
- Osteomyelitis of the foot without diabetes mellitus.
- Studies not available in English

### Search strategy

A comprehensive search strategy will be undertaken from the inception of the databases to 30^th^ April 2022 to identify all published full text studies for inclusion based on the eligibility criteria. Studies included will be subject to their availability in the English language. The following databases will be searched: Medline, PubMed, AMED, the Cochrane Library, Joanna Briggs Institute, CINAHL plus, ProQuest Central, Science Direct, Scopus, Web of Science, ClinicalTrials.gov and ISRCTN Registry. Further studies and grey literature will be retrieved from Google Scholar, ProQuest Dissertations and Thesis Open, ProQuest Central and by hand searching reference lists. There will be no restrictions on publication date. Key search terms will be used alone or in combination including: "Diabetes Mellitus", "Ulcer", "Infection", "Osteomyelitis", "Foot", "Antibiotic(s)" and "Antimicrobial". See additional file 1.

### Study Selection

All quantitative studies for potential inclusion will be based on the eligibility criteria and reviewed by two authors (AU/DR) and documented using PRISMA [38] flow diagram. Studies will then be assessed for their quality by two authors (AU/DR). All data will be imported into the Rayyan Application for blind screening (https://rayyan.qcri.org/welcome). Duplicates will be automatically removed using this software. Two investigators (AU/DR) will blindly screen and cross check the titles and after irrelevant literature has been removed, an abstract review will be completed. Once further irrelevant literature has been excluded, then the full text of the studies identified as being potentially eligible for inclusion will be assessed against the inclusion/exclusion criteria. Any articles that are not available as full text and not available from an institutional library will be sought by contacting the relevant corresponding author to respond within 6 weeks period of the data extraction process. If this is unsuccessful, then the article will be excluded. If a full text article is not available in English, it will be excluded. Any disagreement will be discussed until an agreement is made. In the event there is failure to reach agreement a third reviewer will assist to reach agreement (DS).

### Data Extraction

Data extraction will be completed independently by two reviewers (AU/DR). Any disagreements will be resolved through discussion. A standardised data extraction table will be developed and used for the selected articles. This process will be pilot tested prior to commencing the study. The main fields for data extraction include the effectiveness of systemic antibiotic intervention on DFO including; blood tests; biomarkers including procalcitonin (PCT); radiological imaging; ulcer healing period; limb preservation; no recurrence of osteomyelitis; outcome grades/score; Acute kidney injury (AKI); Minor or major amputation; Mortality. Researchers of studies will be contacted by email to obtain any missing information.

### Quality Assessment

All reviewers will be allocated a divided number of selected studies to independently check for inclusion to reduce bias. All studies will be assessed for their quality. The Cochrane risk-of-bias (RoB 2) [39] tool will be used for assessing bias for all randomised controlled trials. The Newcastle–Ottawa Scale [40] (NOS) will be used for non-randomised controlled trials. Two reviewers (AU/DR) will independently assess the quality of the selected studies.

### Analysis

#### Descriptive Analysis

A narrative synthesis of outcomes will be presented in table format and include:

- Subject group(s) age, sex, ethnicity, location
- Oral or systemic antibiotic intervention
- Antibiotic(s) and dosages/intervals
- Control group or sample size
- Intervention outcomes: blood tests/biomarkers, radiological imaging, healing of ulcer, limb preservation, no recurrence of osteomyelitis, outcome grades/scores used, Acute kidney Injury (AKI), Minor or major amputation and Mortality

### Statistical Analysis

The study interest is in the effectiveness of systemic antibiotic therapy treating osteomyelitis of the foot in subjects with diabetes mellitus. The effectiveness of systemic antibiotics treating infection is measured in different ways amongst medical physicians and surgeons. It is unlikely all studies will be reporting the same design, intervention, and outcome measures therefore the mean change cannot be calculated pre and post systemic antibiotic intervention.

Data synthesis will be conducted, and meta-analysis undertaken using RevMan 5.4.1 Meta-analysis software (Copenhagen: The Nordic Cochrane Centre, The Cochrane Collaboration, 2014). Meta-analysis will be conducted using variables related to clinical resolution of DFO within the individual study period. In the event where there is missing outcome data a missing data analysis will be conducted [41]. The studies will also be grouped by oral versus intravenous administration and separate meta-analyses conducted. Where meta-analysis is not possible due to heterogeneity across the studies a narrative synthesis methodology will used to allow the data to be organized, explored, and presented in a logical way and uncover potential similarities and differences, associations, and patterns within the results.

## Discussion

Multiple studies have reported on the management of DFO with systemic antibiotics [16, 18, 32–34, 42]. This systematic review protocol will synthesise the existing evidence on the effectiveness of systemic antibiotic therapy for treating DFO. The study will identify the diagnostic imaging, laboratory investigations and clinical measures currently used to determine the effectiveness of systemic antibiotic therapy. The findings will summarise existing research and standards in practice for treating DFO with systemic antibiotic therapy. The study may discover if guidelines are required to standardise practice when treating DFO with systemic antibiotic therapy. To the best of our knowledge, this systematic review will be the first to evaluate the effectiveness of systemic antibiotic therapy when treating DFO in adults with diabetes mellitus. The systematic review will not provide specific information on the most single appropriate antibiotic drug or regime for treating DFO. The findings will compliment scientific evidence in support or against systemic antibiotic therapy in the management of DFO worldwide.

## Supplementary Information

**Additional file 1.**

## Data Availability

Not applicable.

## Abbreviations

DFU: Diabetic Foot Ulcer
DFO: Diabetic Foot Osteomyelitis
DFI: Diabetic Foot Infection
IDSA: Infectious Disease Society of America
IWGDF: International Working Group on the Diabetic Foot
PTB: Probe-To-Bone
MRI: Magnetic Resonance Imaging
PET: Positive Emission Tomography
CT: Computed Tomography
SPECT: Single-Photon Emission Computerized Tomography
WBC: White Blood Cell
PCT: Procalcitonin
AKI: Acute Kidney Injury
NOS: Newcastle–Ottawa Scale
PRISMA: Patient Reporting Items for Systematic Reviews and Meta-Analysis
MDRO: Multiple Drug Resistance Organisms
Anti-G-mAb: Anti-Granulocyte Antibodies
PAD: Peripheral Arterial Disease
CLTI: Critical Limb Threatening Ischaemia
RoB: Risk of Bias
