# Supplementary material for: The effectiveness of systemic antibiotics for osteomyelitis of the foot in adults with diabetes mellitus: a systematic review protocol
Source: J Foot Ankle Res. 2022 Jun 17;15:48. doi: 10.1186/s13047-022-00554-3 (PMC9204875; doi:10.1186/s13047-022-00554-3)
Supplement: Supplementary file 1 — Additional file 1. [file 13047_2022_554_MOESM1_ESM.docx]

**Additional file 1: Search strategies examples for Ovid Medline, Medline via EBSCO & Google Scholar.**

Key words: Osteomyelitis, Bone, Bone disease, Diabetes Mellitus, Diabetic foot, Antibiotic, Oral, Intravenous, Infection, Foot, Feet.

**A) Ovid Medline:**

| Search Number | Search |
| --- | --- |
| 1 | exp Diabetes Mellitis/ |
| 2 | Diabet*.ti,ab. |
| 3 | 1 OR 2 |
| 4 | Osteomyel*.ti,ab. |
| 5 | Osteomyelitis/ |
| 6 | 4 OR 5 |
| 7 | (foot or feet or toe* or ankle*).ti,ab. |
| 8 | exp Foot/ |
| 9 | 7 OR 8 |
| 10 | (antibiotic* or antimicrob*). ti,ab. |
| 11 | exp Anti-Bacterial Agents/ |
| 12 | 10 OR 11 |
| 13 | 3 AND 6 AND 9 AND 12 |

**B) Medline via EBSCO search terms:**

| Search Number | Search |
| --- | --- |
| 1 | (MH "Diabetes Mellitus+") |
| 2 | TI Diabet* OR AB Diabet* |
| 3 | 1 OR 2 |
| 4 | TI Osteomyel* OR AB Osteomyel* |
| 5 | (MH "Osteomyelitis") |
| 6 | 4 OR 5 |
| 7 | TI (foot or feet or toe* or ankle*) OR AB (foot or feet or toe* or ankle*) |
| 8 | (MH "Foot+") |
| 9 | 7 OR 8 |
| 10 | TI (antibiotic* or antimicrob*) OR AB (antibiotic* or antimicrob*) |
| 11 | (MH "Anti-Bacterial Agents+") |
| 12 | 10 OR 11 |
| 13 | 3 AND 6 AND 9 AND 12 |
| 14 | Limit 13 to Language: English and Age related: adults 18+ |

**C) Google scholar search terms:**

| **Search terms** | (Osteomyelitis OR Bone OR “Bone disease”) “Diabetes Mellitus” (“Diabetic foot” OR Foot OR Feet) (Antibiotic OR Oral OR Intravenous OR Infection) |
| --- | --- |
